# Exploring the push-pull factors influencing parenting efficacy of fathers of children with ASD: a fuzzy set qualitative comparative analysis

**DOI:** 10.3389/fpsyt.2024.1303484

**Published:** 2024-05-08

**Authors:** Linli Zhu, Xinbin Ge, Weijun Huang, Leyi Shao, Xiaolan Ma

**Affiliations:** ^1^ School of Education, South China Normal University, Guangzhou, Guangdong, China; ^2^ School of Educational Science, Nanning Normal University, Nanning, Guangxi, China; ^3^ School of Special Education, Guangxi College for Preschool Education, Nanning, Guangxi, China; ^4^ Graduate School, Lyceum of the Philippines University, Batangas, Philippines

**Keywords:** parenting efficacy, father, ASD, push-pull factors, fuzzy-set qualitative comparative analysis (fsQCA), China

## Abstract

**Objectives:**

This study aimed to explore the core factors and configurations influencing parenting efficacy for fathers of children with ASD in Western China by using a configuration analysis.

**Background:**

Understanding the push-pull factors related to parenting efficacy for fathers raising children with ASD can support the fathers of children with ASD to participate in education affairs and improve the quality of family education, which is of significant importance to both individuals and society. This study recruited 156 fathers of children with ASD from China.

**Results:**

The results suggested that there was more than one (sub) configuration for achieving a high level of parenting efficacy among fathers of children with ASD. (b) The configurations could be summarized into three main types as follows: “working within the system*flexible working time,” “high level of education*high level of other support,” and “severe disability*fewer interaction with wife.” (c) The two variables, “family income” and “community support,” were not as important as they were shown in previous studies, and their effects often depended on how they interact with other variables.

**Conclusions:**

Findings highlighted the validity of deploying configuration analysis (based on the ecosystem theory) to establish the relationship between independent variables and the parenting efficacy of fathers of children with ASD. The discovery of more than one configuration led to a new consensus on how multiple factors influence parenting efficacy. Implications were suggested for practitioners, policymakers, and future research alike.

## 1 Introduction

In 2021, China promulgated the Law on the Promotion of Family Education, which clarified the responsibility of parents as the main body of family education. As an important national strategy in China, family education has been raised to an unprecedented level, and the issue of parental education for children with ASD has been increasingly emphasized in the field of special education. Autism spectrum disorder (ASD) is a group of mental developmental disorders that mainly manifests itself in social disorders and stereotypical behaviors, and it starts before the age of 3 years, with a predominance of boys (1). Reports on the prevalence of ASD vary from country to country. Although there is a lack of epidemiological surveys in large samples, there is an overall increasing trend (2). Parents of children with ASD have higher levels of stress and lower parenting efficacy than parents of typically developing (TD) children and other children with disabilities (3–8). Parenting efficacy has a significant impact on parenting. More research is needed in the area of parenting efficacy to better parent children diagnosed with ASD. However, perhaps due to the traditional Chinese concept of gender roles, mothers are often viewed as the primary caregivers of children, playing the emotional role of “gatekeepers,” while fathers play the instrumental role of “breadwinners,” creating a “male-dominated, female-dominated” family environment. “Therefore, the discussion of parental efficacy in children with ASD is often limited to the mother’s role, and most of the research data is collected from mothers, so we know very little about the fathers of children with ASD (9, 10). The development of families of children with ASD is based on the full utilization of parenting roles, which should not be limited to mothers, and the changing socio-economic structure and values, which require more attention to the parenting efficacy of fathers.

In addition, previous studies on parenting efficacy had relied on methods such as multiple regression analysis and structural equation modeling to calculate the net effects of potential variables on specific outcomes; however, these studies might have methodological problems in dealing with the combined effects of complex variables and might fail to explore the effects of the interactions and matches between multiple factors on parenting efficacy, but only explored the “net effect” of independent variables on parenting efficacy. Net effect analysis focuses on the contribution of a single independent variable to the explanation of the dependent variable and has the advantage of explaining the variability of the variables, but the parenting efficacy of fathers of children with ASD is not the result of the independent action of a single variable, but rather the effect of an interactive combination of multiple variables. In this regard, the Fuzzy-Sets Qualitative Comparison Approach (Fuzzy-Sets QCA, fsQCA) is advantageous. FsQCA is a methodology that has emerged in recent years and is now being used in many fields, including psychology and education (11–13). It relies on Boolean logic to realize the logical principle of comparisons between multiple samples and is a set-theoretic approach to causal analysis based on the premise that outcomes tend to be caused by more than one factor, rather than by any single factor (14). Considering that the subject of this study was the parenting efficacy of fathers of children with ASD, which was characterized by “multiple complex concurrent causation”, fsQCA was a very appropriate choice to explore how the synergistic effects of different factors affected the parenting efficacy of fathers of children with ASD.

## 2 Research background

### 2.1 Parenting efficacy

Parenting efficacy was derived from the concept of “self-efficacy” in Bandura’s Social Learning Theory, which Bandura (1994) defined as an individual’s belief in the efficacy of his or her own abilities (15), so parenting efficacy referred to a parent’s beliefs about parenting and was a subjective judgment about the ability to successfully engage in parenting behaviors that affected the health and development of the child. The concept of parenting efficacy varies depending on the focus of the study and includes parenting skills, nurturing skills, emotional communication, parent-child interaction, and conflict-resolution efficacy. This study focused on the parenting efficacy of fathers of children with ASD, so parenting efficacy in this study referred to a belief that fathers of children with ASD when practicing parenting, were satisfied with their performance in their parenting roles, felt confident in nurturing efficacy, emotional expression and communication efficacy, and self-affirmation, and were happy in dealing with parenting matters.

Most of the literature on the parenting efficacy of children with ASD has discussed the parenting efficacy of mothers (16–19), and most of its investigations have continued to focus on mothers, with limited direct research on the parenting efficacy of fathers of children with ASD. Even Bandura’s work rarely mentioned the parenting efficacy of fathers alone.

In general, existing research on parenting efficacy mainly involved the following: (a) The influencing factors of parenting efficacy included the type of occupation (20), social support (21–23), family economic level (24–26), parents’ education level (27), couples’ interaction, and communication patterns (28, 29); (b) The mediating role of parenting efficacy suggested that enhancing parenting efficacy could positively influence young children’s social competence (30) and reduce problem behaviors in young children, which could indirectly reduce parenting stress and improve the quality of family life (31); (c) Correlational studies of parenting efficacy mainly explored the correlation between parenting efficacy and parenting stress (32, 33), the correlation between parenting efficacy and parental involvement (34, 35); (d) The effect of parenting efficacy was found to predict parenting outcomes (36), facilitate early intervention and development for children with special needs (37), and the parenting efficacy of fathers was also predictive of father involvement (38).

In addition, a research study has pointed out that if the children in the family are healthy, the parental efficacy is higher; however, if the children in the family are developmentally delayed or disabled, the parenting efficacy of the parents is lower (37). It is difficult for parents who are expecting a new baby to be psychologically prepared to deal with their child’s disability, and the first reaction of fathers of children with ASD is overwhelming denial and skepticism when confronted with their child. Parenting a child with ASD involves a lot of challenges, which make fathers’ parenting efficacy experience the impact. Therefore, the degree of disability of the child with ASD also needs to be considered as an antecedent condition when constructing the conceptual model for this study.

### 2.2 Ecosystem theory

In the late 1970s and early 1980s, scholars began to perceive the systemic character of the family, in which fathers were no longer regarded as isolated individuals in the family but as members of the network of family members’ interactions, and a variety of theoretical perspectives were developed, such as ecosystem theory, family systems theory (39). Among the various theories, the most complete doctrine that explains the interaction between the individual and the environment is the ecosystem theory proposed by Bronfenbrenner, which emphasizes the influence of multiple environments on human behavior and development, and understands the interaction between the individual and the environment in terms of the individual and the different hierarchical systems in the environment in which he/she is embedded. In the ecosystem model, the individual is placed in the very center, from the inside out, and each system is interlocked, containing microsystems, mesosystems, exosystems, and macrosystems. Relevant factors that may affect the parenting efficacy of fathers of children with ASD analyzed from the ecosystem theory are described as follows: (a) Microsystem: a system in which individuals are directly involved. Factors in the microsystem that may directly affect the parenting efficacy of fathers include fathers’ personal factors (e.g., educational level, working hours, occupational category), spousal factors (e.g., husband-wife interactions), and children’s factors (e.g., gender, disability); (b) Mesosystems: refers to the interaction of two or more small systems in which fathers are directly involved, such as family-school, family-neighborhood, small family-extended family and family-workplace; (c) Exosystem: It is a system in which fathers are not directly involved, but which affects the microsystems, thus indirectly affecting personal growth and values; influences in the exosystem may include community networks, communication media, legal services, etc. (d) Macrosystem: It refers to the influence of political, cultural, economic and social factors on the attitudes and values of fathers (40).

The ecosystem theory takes a systemic perspective to explore how family roles are influenced by various factors, asserting that individuals and families cannot be insulated from the influences of the external environment. This illustrates the extent to which family ecology is affected when a child has ASD. However, ecosystem theory does not emphasize a simple linear relationship between single variables; its theoretical constructs instead prioritize the relationships from the subtle individual level to the macro-social context (41). Therefore, when investigating parenting efficacy based on ecosystem theory, it is essential to go beyond examining the relationship between fathers and their families and to analyze the interactions among the major factors within the entire ecosystem.

This study aimed to investigate the parenting efficacy of fathers with children diagnosed with ASD. Fathers of children with ASD exist within the macro-social context and interact with their own families and organizations, thus experiencing the influence of various elements within the ecosystem. Consequently, ecosystem theory provides an appropriate theoretical framework for analyzing the intricate mechanisms that shape the parenting efficacy of fathers of children with ASD. In this paper, fathers were considered as central figures within the ecosystem, and the microsystems, mesosystems, ecosystems, and macrosystems of ecosystem theory were simplified into individual factors, intra-familial factors, and extra-familial factors.

### 2.3 Research gap

Parenting efficacy exerts a significant influence on parenting practices. There is a growing need for further research in the realm of parenting efficacy; however, existing studies predominantly concentrate on mothers, often overlooking the perspective of fathers. It is unequivocal that the parenting efficacy of fathers with children diagnosed with ASD results from a multifaceted interplay of various factors. Nonetheless, the manner in which these diverse factors interact to shape the parenting efficacy of fathers in this context remains an open question. Presently, much of the research on parenting efficacy relies on methodologies such as multiple regression analysis and structural equation modeling. The impact of individual factors is typically determined while keeping all other variables constant, employing regression-based analytical techniques. Despite efforts to explore the interrelationships among these factors, through the inclusion of interaction terms or instrumental variables, capturing the intricate dynamics among multiple factors, especially those exceeding three, remains a formidable challenge for regression estimation (42, 43).

Given that it is essential to assess the impact of any factor in conjunction with other variables or take into account how changes in other factors coincide with changes in the focal factor, this study recommends employing fsQCA, a configuration analysis method, to explore the influences of individual factors on paternal parenting efficacy in the context of children with ASD.

## 3 Research model

**Figure 1:**
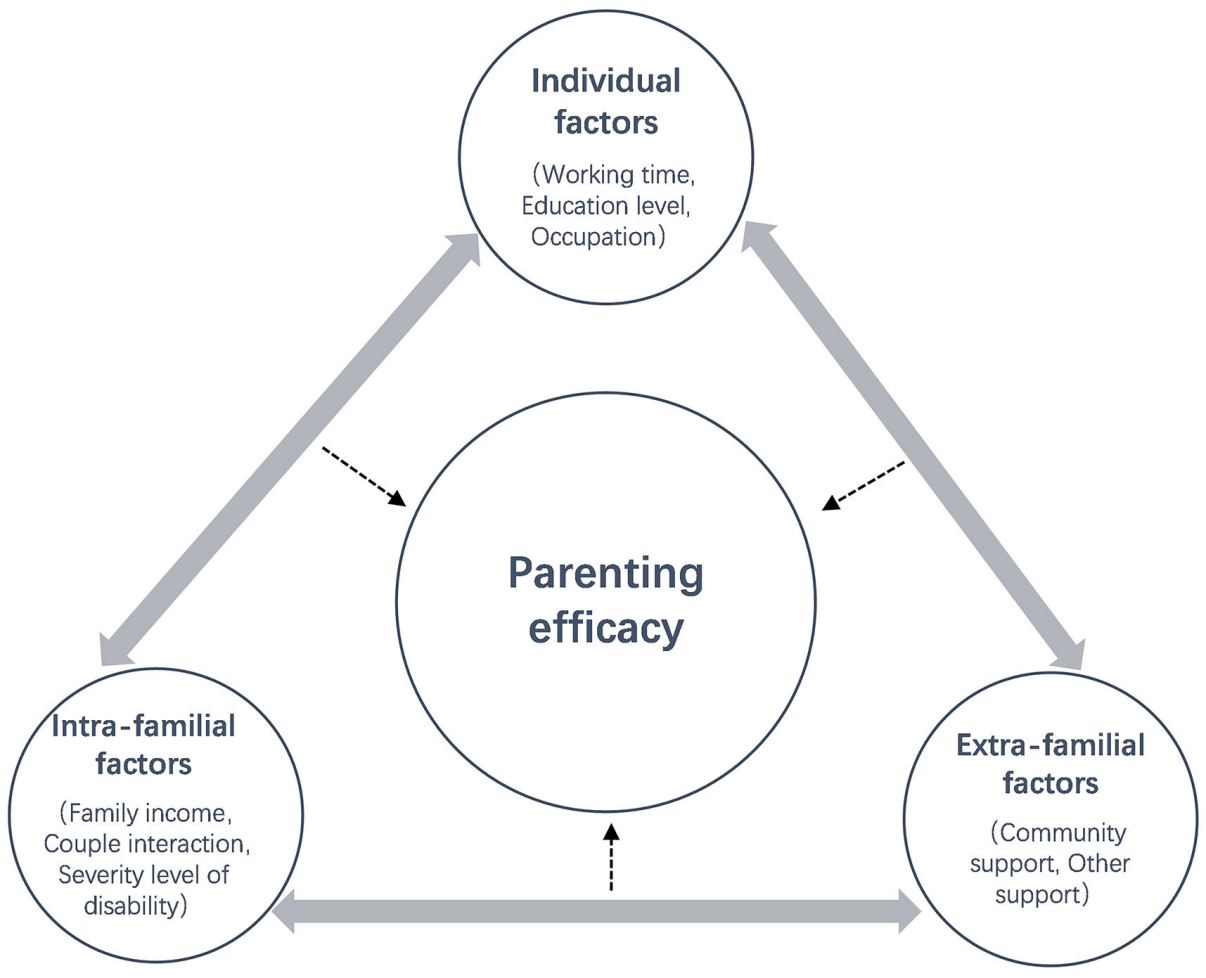
Conceptual model of parenting efficacy of fathers of children with ASD.

Grounded in ecological systems theory, this study posited a hypothesis that the parenting efficacy of fathers with children diagnosed with ASD was influenced by a combination of various individual, intra-familial, and extra-familial factors. It constructed a conceptual model encompassing one outcome variable and eight independent variables. Specifically, the outcome variable was parenting efficacy (PE) of fathers of children with ASD, while the independent variables comprised working time (WT), education level (EDU), occupation (OCC), family income (FI), couple interaction (CI), severe level of disability (SLD), community support (CS), and other support (OS). Consequently, this study introduced the following conceptual model (see **Figure 1**):

PE = f (WT, EDU, OCC, FI, CI, SLD, CS, OS)

## 4 Research methodology

### 4.1 Method of analysis

Fuzzy-set qualitative comparative analysis (fsQCA) is a mixed-method approach developed by Ragin (2008). The method relies on Boolean logic to realize the logical principle of comparisons between multiple samples and is a set-theoretic approach to causal analysis based on the premise that outcomes tend to be caused by more than one factor, rather than by any single factor (14, 42). It breaks through the limitations of the sample size and allows for a more in-depth configuration analysis of the results of samples of different sizes, and it also breaks through the limitations of dichotomous variables and enters the field of interval variables, so it has a wider scope of application and is more conducive to analyzing the relationship between variables with fuzzy characteristics. Considering that the subject of this study was the parenting efficacy of fathers of children with ASD, which was characterized by “multiple complex concurrent causation”, fsQCA was a very appropriate choice to explore how the synergistic effects of different factors affected the parenting efficacy of fathers of children with ASD. Therefore, this study used fuzzy-set qualitative comparative analysis on a sample of 156 fathers having children with ASD in Western China to provide an understanding of the push-pull factors influencing parenting efficacy of fathers of children with ASD.

Configuration analysis using fsQCA involves five primary steps: defining variables, which encompass both dependent and independent variables, and constructing a conceptual model; collecting sample data; calibrating the data; creating truth tables and processing the data; and performing configuration analysis (12).

### 4.2 Data collection

In this study, we conducted a survey involving fathers of children diagnosed with ASD. Questionnaires were distributed to 194 families who had children with ASD in China. A total of 158 fathers completed the questionnaires in their entirety, while 36 fathers dropped out of participation midway through the program, citing that “the children were primarily cared for by their mothers, and I possessed limited knowledge on the subject.” After eliminating questionnaires displaying evident patterns or those that were completed unusually quickly, we obtained 156 valid responses, yielding a validity rate of 98.73%. The sample size acquired in this study aligns with the requirements of the fsQCA method for medium-sized samples (44). The demographic information was shown in **Table 1**.

**Table 1:** Demographic information of fathers and children with ASD.

Fathers	*n* (%)	Children with ASD	*n* (%)
**Working time**		**Gender**	
Fixed working time	88 (56%)	Male	130 (83%)
Flexible working time	68 (44%)	Female	26 (17%)
**Education level**		**Age**	
High school or below	22 (14%)	1~3	102 (65%)
Vocational and technical school	36 (23%)	4~6	38 (24%)
Bachelor’s degree	66 (42%)	7~9	12 (8%)
Master’s degree or above	32 (21%)	10~12	4 (3%)
**Couple interaction**		**Severity level of disability**	
Never discuss parenting matters	4 (3%)	Mild	78 (50%)
Rarely discusses parenting matters	14 (9%)	Moderate	68 (44%)
Occasionally discuss parenting matters	58 (37%)	Severe	10 (6%)
Often discuss parenting matters	80 (51%)	Extremely severe	0 (0%)
**Occupation**			
Working within the system	36 (23%)		
Working outside the system	120 (77%)		

### 4.3 Measurements checking

The questionnaire utilized in this study was adapted from a Short-Form Parenting Self-Efficacy Scale developed by Yao (2010) and Wu (2014) (45, 46). To enhance its relevance to the field of special education and ensure that research participants could comprehend the scale’s content with greater clarity and accuracy, the original questionnaire underwent necessary modifications based on interviews conducted with pre-survey respondents. Here is an explanation of which dimensions have undergone modifications: (a) In the section on basic personal information, the options for “Your current occupation” have been changed from some specific occupations to “Working within the system” and “Working outside the system”; (b)In the “Basic Information” section, we have added a new question, which asks “What is the severity level of your child’s disability?” (c)In the “Personal Feelings” section, we have described the child’s type of disorder as ASD. This refined version was then employed as the official questionnaire for the study.

The overall Cronbach’s α coefficient for the questionnaire was 0.894, with each variable demonstrating a Cronbach’s α exceeding 0.8, indicating acceptable internal consistency. Structural validity was assessed through factor analysis, yielding a Kaiser-Meyer-Olkin (KMO) value of 0.80 and a minimum cumulative variance contribution rate of 73.03%. Furthermore, all question items displayed factor loadings exceeding 0.50, affirming the questionnaire’s robust measurement validity.

### 4.4 FsQCA

#### 4.4.1 Data calibration

When conducting the fsQCA analysis, each causal condition and outcome is treated as a set, and every sample is assigned a corresponding membership in these sets. This involves converting the data of the causal condition (independent variable) into fuzzy set memberships by defining three anchor points. Calibration is the process of recoding variables into fuzzy sets and assigning set membership to individual samples (44). Data calibration is typically carried out using either direct or indirect methods. In direct calibration, three anchor points are established to determine the degree of membership in the fuzzy set for each case: full membership (scored as 0.95), full non-membership (scored as 0.05), and the crossover point (scored as 0.50) (43). In contrast, indirect calibration involves adjusting measurements based on the researcher’s expertise or theoretical considerations following a qualitative assessment.

In this study, we employed a combination of direct and indirect calibration methods to transform the data into fuzzy set memberships. Specifically, as illustrated in **Table 2**: (a)Variables such as “working time” and “occupation” were dichotomous (2-category) variable. Concerning the variable “working time,” a “fixed working time” was assigned a value of 1, while “flexible working time” was assigned a value of 0. For the variable “occupation,” a value of “working within the system” was designated as 1, while “working outside the system” was marked as 0; (b) Variables such as “education level,” “family income,” “couple interaction,” and “severity level of disability” were tetrachoric (4-category) variables. Referring to Wan’s (2019) assignment of values to the tetrachoric (4-category) variables, this study could assign values of 0, 0.6, 0.8, and 1 according to the level from lowest to highest (47); (c) In the absence of available external standards or benchmarks for other variables, we calibrated their data using the upper quartile value (for full membership), lower quartile value (for full non-membership), and median value (for the crossover point) (42).

**Table 2:** Data calibration for dependent variable and independent variables.

	Type	Name	Description for variables	Value	Anchors
Independent variables	Individual factors	Working time (WT)	Fixed working time Flexible working time	1 0	—
		Education level (EDU)	High school or below Vocational and technical school Bachelor’s degree Master’s degree or above	0 0.6 0.8 1	—
		Occupation (OCC)	Working within the system Working outside the system	1 0	—
	Intra-familial factors	Family annual income (FI)	below 40,000 RMB 40,000~80,000 RMB 80,000~120,000 RMB over 120,000 RMB	0 0.6 0.8 1	—
		Couple interaction (CI)	Never discuss parenting matters. Rarely discusses parenting matters Occasionally discuss parenting matters Often discuss parenting matters	0 0.6 0.8 1	—
		Severity level of disability (SLD)	Mild Moderate Severe Extremely severe	0 0.6 0.8 1	—
	Extra-familial factors	Community support (CS)	Community support includes support from child’s grandparents, neighbors, co-workers, relatives and other ASD parent groups	—	3.83 3.58 3.17
		Other support (OS)	Other support includes religious beliefs, social welfare, education policies, medical resources, etc.	—	3.44 2.75 2.00
Dependent variable	—	Parenting efficacy (PE)	—	—	4.17 3.96 3.62

“Working within the system” refers to employment within China’s state entities, including state organs, state-owned enterprises, and institutions, among others.

#### 4.4.2 Truth table establishment and refinement

A truth table lists all possible combinations of conditions expressed as binary states. Constructing the truth table means identifying the conditional combinations of the samples and their corresponding associations with the results based on the fuzzy set membership scores calibrated to the data. Since fsQCA calculates all logical combinations, there will be many combinations with zero frequency, and the truth table needs to be refined by setting frequency cutoff and consistency threshold. It has been argued that a consistency threshold of 0.75 is the minimum acceptable value, and the frequency cutoff should be able to capture more than 80% of the cases assigned to the configuration (14). Therefore, in this study, the frequency cutoff was set at one, and the consistency threshold was set at 0.8, and was used to refine the truth table.

#### 4.4.3 Configuration analysis

The process of configuration analysis encompassed the evaluation of set and subset relationships using the refined truth table, employing the Quine-McCluskey algorithm in conjunction with counterfactual analysis (42, 43). The Quine-McCluskey algorithm identified condition combinations that consistently resulted in the specific outcome while simultaneously excluding conditions that only sporadically influenced the outcome. In this study, configuration analysis was conducted to ascertain the presence of a high level of parenting efficacy utilizing the fsQCA software.

### 4.5 Complementary analysis

#### 4.5.1 Sensitivity analysis

Sensitivity analysis was performed to assess the resilience of the findings through the utilization of alternative condition specifications. Specifically, it was examined by modifying the consistency threshold (14, 42). In this study, sensitivity analysis was executed by varying the consistency thresholds from 0.8 to 0.78 and 0.82, while keeping all other parameters constant.

#### 4.5.2 Predict validity analysis

Predictive validity analysis was employed to assess the hypothesis configuration model’s ability to forecast the outcome variable across various datasets (48). The original dataset was randomly divided into two equal sub-samples: a modeling sub-sample (Sub-sample 1) and a holdout sub-sample (Sub-sample 2). FsQCA was conducted on Sub-sample 1, utilizing the same frequency cutoff and consistency threshold applied in the primary analysis (49). Subsequently, the configuration model derived from Sub-sample 1 was tested on Sub-sample 2 to evaluate its consistency and coverage performance in comparison to Sub-sample 1 (49).

#### 4.5.3 *Post hoc* analysis

Incorporating fsQCA solutions within a regression framework via Tobit regression analysis can be regarded as a *post hoc* examination, providing supplementary perspectives into the phenomenon under examination (50). Consequently, this study conducted Tobit regression analyses with parenting efficacy as the dependent variable, both individually and with all configural statements concerning the level of parenting efficacy as independent variables. The values of the configural statements were transformed in accordance with Boolean logic rules based on their original values (50).

## 5 Results

### 5.1 FsQCA results

Configuration analysis results in three types of solutions: complex solution (without the use of “logical remainder”), intermediate solution (using “logical remainder” that is consistent with theory and practice), and parsimonious solution (using all “logical remainder” that may help to simplify the configuration, without evaluating its rationality). The “solution” is a combination of conditions supported by a large number of cases. Complex solutions present all possible combinations of conditions, which may be large in number, and are therefore automatically simplified into parsimonious, intermediate “solutions”. Intermediate solutions do not parsimoniously remove the necessary conditions, so it is often considered to report intermediate solutions in the paper and to distinguish between core conditions and peripheral conditions in combination with parsimonious solutions (14). The core conditions are variables that are strongly causally related to the outcome, and peripheral conditions are variables that are weakly causally related to the outcome (42). If the causal conditions appear in both the parsimonious and intermediate solutions, they are the core conditions; if these conditions appear only in the intermediate solution, they are the peripheral conditions. There are six configurations of high parenting efficacy through fsQCA (see **Tables 3**, **4**). The overall consistency of the configurations was 0.866, showing a high degree of consistency, indicating that the six configurations were sufficient to result in high parenting efficacy; the coverage of the solutions was 0.293, indicating that they can explain 29% of the sample.

**Table 3:** Results of the configuration analysis.

Solutions	Parenting Efficacy
Parsimonious solution	~WT*OCC ~WT*EDU*~CS*OS ~CI*SLD WT*~FI*~SLD EDU*~OCC*SLD*~CS*OS
Intermediate solution	~WT*OCC*~CS*~OS ~WT*EDU*~CS*OS WT*~FI*~SLD*~CS*~OS EDU*~OCC*SLD*~CS*OS ~WT*OCC*SLD*CS*OS ~WT*~FI*~CI*SLD*~CS*OS

*Means Boolean logic “and”; ~means Boolean logic “not”.

**Table 4:** Configurations for achieving high parenting efficacy.

Factor	Configuration					
	1a	1b	2	3	4	5
Working time (WT)	⨀	⨀	⨀	●		⊙
Education level (EDU)			●		●	
Occupation (OCC)	●	●			⨀	
Family income (FI)				⨀		⊙
Couple interaction (CI)						⨀
Severity level of disability (SLD)		•		⨀	●	●
Community support (CS)	⊙	•	⨀	⊙	⨀	⊙
Other support (OS)	⊙	•	●	⊙	●	•
Raw coverage	0.037	0.020	0.072	0.081	0.096	0.033
Unique coverage	0.036	0.013	0.051	0.063	0.064	0.027
Consistency	0.851	1	0.879	0.817	0.927	0.806
Solution coverage	0.293					
Solution consistency	0.866					

(a) ● indicates the presence of a condition and ⨀ indicates its absence. Large circles indicate core conditions. Small circles indicate peripheral conditions. Blank spaces indicate that the condition is indifferent. (b) The two complex solutions for a high level of parenting efficacy are sub-configurations (we later name them Configurations 1a and 1b) that were formed based on the same set of core factors (~WT*OCC).

In order to facilitate the interpretation of the results and visualize them, the parsimonious and intermediate solutions could be combined to create a table containing the core and periphery conditions. As can be seen in **Table 4**, there were six configurations that achieved high parenting efficacy. The peripheral conditions of configuration 1a were community support and other support, and those of configuration 1b were severity level of disability, community support and other support; the core conditions common to both configurations 1a and 1b, namely, “flexible work time” (WT) and “working within the system” (OCC), indicated that fathers of children with ASD who worked within the system and had flexible work time had higher parenting efficacy. Configuration 2 indicated that fathers of children with ASD with high levels of education were able to increase their parenting efficacy if they received insufficient community support, but had flexible work hours and adequate other support. Configuration 3 indicated that fathers of children with ASD had higher parenting efficacy if they had flexible work time and their children’s disabilities were mild when the community and other support were limited and the family income was low. Configuration 4 indicated that fathers of children with ASD who were highly educated and worked outside the system were able to achieve higher parenting efficacy when other support was adequate, even if their children’s disabilities were severe and the community support was inadequate. The core conditions of configuration 5 were “severe disability” (SLD) and “fewer interactions with wife” (~CI), which indicated that when a child with ASD had severe disability, the father’s parenting efficacy was enhanced by fewer interactions with the mother.

By cross-sectional comparative analysis between the configurations, the following results could be obtained: first, configurations 1a and 1b, which achieve high parenting efficacy, had the same core conditions, i.e., “flexible working time” and “working within the system”; and configurations 2 and 4 had the same core conditions, i.e., “low level of community support”, “high level of education”, and “high level of other support”. Second, the variables of “working time”, “occupation”, and “severity level of disability” were more frequently emphasized as core conditions in the configurations for achieving high parenting efficacy. Third, the four variables of “working time”, “family income”, “couple interaction”, and “community support” mostly appeared as opposite values in the configurations, indicating that they needed to be combined with other variables to be more effective.

### 5.2 Complementary analysis results

#### 5.2.1 Sensitivity analysis result

The results of the qualitative comparative analysis may be sensitive to the calibration strategy, consistency threshold and frequency cutoff (51). That is, sensitivity analysis can be performed by adjusting the calibration strategy/consistency threshold/frequency cutoff. In this study, sensitivity analysis was conducted by adjusting the consistency threshold, which was sequentially adjusted from 0.8 to 0.78 and 0.82, while keeping the others constant, and the configuration analysis was conducted again. The results indicated a small change in the number of solutions for parenting efficacy, but the change was not sufficient to support meaningful and distinct explanations. Therefore, the findings of this study remained robust.

#### 5.2.2 Predictive validity result

**Figure 2:**
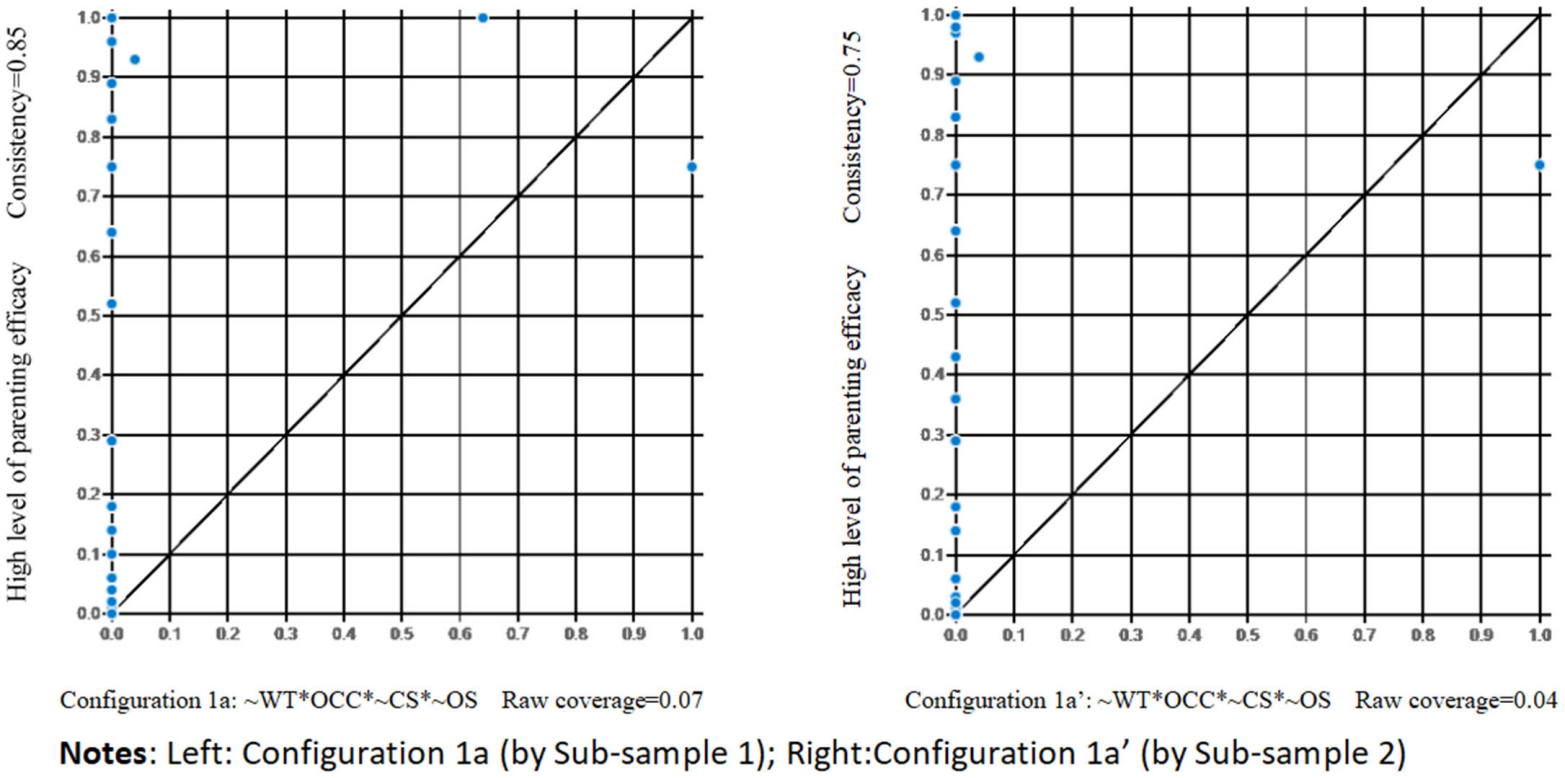
XY scatter plots of parenting efficacy in Configurations 1a and 1a’.

Configurations in Sub-sample 1 underwent testing using Sub-sample 2 data. All model tests yielded comparable consistency and coverage results (**Figure 2** depicts the model testing of Configuration 1a). Thus, the suggested configurations exhibited strong predictive capabilities across diverse data sets.

#### 5.2.3 *Post hoc* analysis result

Results of the *post hoc* analysis (see **Table 5**) show that Configuration 1 had a positively significant effect (*β* = 1.117; *p* = 0.049), as did Configuration 2 (*β* = 1.609; *p* = 0.012), on parenting efficacy. Configuration 3 also had a significant effect on parenting efficacy (*β* = 1.322, *p* = 0.018), as did Configuration 4 (*β* = 1.517, *p* = 0.003), and Configuration 5 (*β* = 1.680, *p* = 0.063). These results correspond roughly to the results obtained by fsQCA.

**Table 5:** Results of the Tobit regression analysis for parenting efficacy.

Independent variable	Coefficient	Standard Error	z-Statistic	Probability
Dependent variable: parenting efficacy (PE)				
Configuration 1	1.117^**^	0.568	1.968	0.049
Configuration 2	1.609^**^	0.641	2.510	0.012
Configuration 3	1.322^**^	0.557	2.373	0.018
Configuration 4	1.517^***^	0.518	2.930	0.003
Configuration 5	1.680^*^	0.905	1.857	0.063

^*^p< 0.1; ^**^p< 0.05; ^***^p< 0.01.

## 6 Discussions

### 6.1 Main findings

The core conditions in the configurations are essential elements that indicate a strong causal relationship between the causal conditions and the outcome, while the peripheral conditions are less important or even expendable and exchangeable (42). Based on this, it was necessary for this study to focus on the core conditions in the configuration, and to analyze the push-pull factors influencing parenting efficacy of fathers of children with ASD in combination with the core conditions. Specific combinations of core conditions could be categorized into the following three typical situations.

Type 1: Work Empowerment. The core condition combination for this type was “working within the system * flexible working time”. The results of the combination analyses showed that “working within the system * flexible working time” was one of the core condition combinations for achieving high parenting efficacy. This may be due to the fact that institutionalized jobs usually provide more stable job security and financial income, which made fathers of children with ASD feel a certain sense of security. So, they could better balance their work and family responsibilities, thus enhancing parenting efficacy. This was consistent with the findings of Fan (2013). He found that fathers who were working within the system (e.g., civil servants, teachers, etc.) spent more time involved in early childhood parenting relative to fathers in other occupations (20). Working within the system also usually provided relatively good welfare and resource, including paid vacations, medical resource, and educational information, etc., which can reduce the burden of fathers of children with ASD and thus enhance their parenting efficacy. In addition, the flexibility of working time was also beneficial to fathers in balancing family and work so as to better raise their children with ASD.

Type 2: Resource Empowerment. The combination of core conditions for this type was “high level of education * high level of other support”. In general, education level cannot directly influence the parenting efficacy of fathers of children with ASD, but it can ultimately improve parenting efficacy by influencing fathers’ attitudes toward learning and positive access to resources (25). Other supports in this study included religious beliefs, social welfare, educational policies, and medical resources, which were part of exosystems and macrosystems in ecosystem theory. Ecosystem theory suggested that individuals cannot be insulated from the influence of the external environment (39, 41), and the parenting efficacy of fathers of children with ASD was also affected by external resources. A high level of other support (adequate external resources) was conducive to enhancing parenting efficacy, and fathers with a high level of education were more active in obtaining support from external resources (25).

Type 3: Family Empowerment. The combination of core conditions for this type was “severe disability *fewer interaction with wife”. The parenting efficacy of fathers of children with ASD was not only the result of exosystem and macrosystem catalyzation but also inextricably linked to the ecology within the family. According to the results of previous studies, for fathers of children with ASD, affected by the severity level of disability of the child with ASD, there would be many dilemmas in participating in parenting affairs. Because of the uncertainty of the etiology of ASD, the complexity of ASD, and the specialization of the rehabilitation training, the fathers lacked the appropriate and effective methods to help their children improve their social abilities and deal with problem behaviors, etc., which will in turn lead to pessimism, doubts about their parenting ability (27). The results of the present study found that if children with ASD had a severe disability and the fathers had fewer interactions with their wives (i.e., fewer discussions about child-rearing matters), the fathers were instead able to achieve a high level of parenting efficacy. This was contrary to Potter (2017). This study argued that the reduced frequency of couple interactions did not represent a discordant couple relationship nor a father’s unwillingness to be involved in parenting matters. This is because in the culture of Chinese society, fathers are often perceived as reticent or stoic, and the display of intimate qualities is seen as a threat to masculine masculinity (52). When the children’s disabilities were severe, their fathers felt a great deal of parenting pressure (37). However, due to the influence of Chinese culture, Chinese fathers seldom communicated with their wives about the pressure, but rather took the burden on their own, reduced the frequency of discussing child-rearing matters with their wives and gave themselves the space to be alone, thus enhancing their parenting efficacy.

In addition, some scholars believed that community support had a significant impact on parenting efficacy. For instance, Sun (2015) argued that community support, which mostly belonged to spiritual support, could mediate the parenting stress and emotions of families of children with ASD and improve parenting efficacy (22); Yao (2018) concluded that the role of informal support (e.g., from friends and neighbors) and formal support (e.g., from organized institutions) even surpassed the role of family factors in the context of family functioning deficits (23). In addition, some studies suggested that family income was the main moderator variable of parenting stress (24) and the main demographic variable influencing parenting efficacy (53). However, the results of this study showed that “family income” and “community support” mostly appeared as their opposing values in the configurations, which proved that these two variables were not as important as they were shown in the previous studies and that their effects were largely dependent on the combination of other variables. Therefore, when exploring the complex causality of parenting efficacy, it was important to consider not only the single effects of family income and community support but also the effects of other causal conditions.

### 6.2 Theoretical implications

This study underscores the importance of prioritizing configurations that lead to high levels of parenting efficacy over the influence of individual factors. By constructing a configuration model incorporating multiple factors (varied independent variables) and parenting efficacy (the outcome variable) within the framework of configuration theory, this study provides deeper insights into the roles of these factors in fathers’ parenting efficacy when raising children with ASD. The findings serve as a foundation for future research aimed at exploring the interactions among multiple factors in the context of paternal parenting efficacy studies. The results indicate the existence of more than one (sub) configuration associated with high parenting efficacy, affirming the presence of multiple pathways to achieving it. These findings contribute to establishing a consensus regarding the influence of various factors on fathers’ parenting efficacy in the context of children with ASD and underscore the applicability of configuration theory in the domain of fatherhood research. Furthermore, the results offer theoretical insights that broaden the perspective of paternal research, enhance the interpretative framework of the ecosystem system, and enrich the methodological toolkit for researching fatherhood in the context of children with ASD.

### 6.3 Practical implications

The personal factors of fathers included working time, education level, and occupation. The results of this study showed that “flexible working time,” “working within the system,” and “high level of education” were important factors affecting the parenting efficacy of fathers of children with ASD and often appeared as the core conditions in configurations. Although “education level” couldn’t directly affect the parenting efficacy of fathers of children with ASD, it was combined with “other support” variables to realize the purpose of improving parenting efficacy. “Flexible working time” also often had an additive effect with “working within the system” to enhance the parenting efficacy of fathers of children with ASD. Although it was difficult for fathers of children with ASD to adjust their jobs, and it was not possible to provide stable employment opportunities for all fathers of children with ASD, it was possible to focus on the impact of the hidden factors of “flexible working time” and “ working within the system” on the fathers of children with ASD, as well as on the needs of the fathers who did not work within the system. In addition, “high levels of other support” among the extra-familial factors also had a positive impact on the parenting efficacy of fathers of children with ASD, which suggests that government departments and social organizations should strengthen their support for fathers of children with ASD, such as by providing stable job security, improving welfare benefits, and enhancing support for social welfare, education policies, and medical resources, so as to enhance fathers’ sense of security and efficacy in caring for and educating their children with ASD.

## 7 Conclusion, limitations and future research

### 7.1 Conclusion

Using a sample of 156 fathers of children with ASD, this study used fsQCA to determine the structure of the parenting efficacy of fathers who have children with ASD and the push-pull factors that significantly influenced it. This study indicated that these factors were critical for a high level of parenting efficacy among fathers of children with ASD. Finally, theoretical implications were provided for a comprehensive understanding of the parenting efficacy of fathers of children with ASD in China, and practical implications were suggested for enhancing the parenting efficacy of fathers of children with ASD.

### 7.2 Limitations and future research

This study only examined the static relationship between the independent variables and the parenting efficacy of fathers of children with ASD (i.e., the dependent variable) and did not consider the dynamic evolution of the independent variable over time. That is, the study provided a static view of parenting efficacy, which could be contrasted with a dynamic approach as a consequence of the adaptive and formative processes depending on the different intervention programmes that were put in place. Therefore, with the continuous development and improvement of the dynamic fsQCA methodology, we may consider comparative analyses of solutions over multiple time periods in the future to explore the trajectories of change. Secondly, although the theoretical model constructed in this study had integrated as many of the eight key causal conditions under the ecosystem framework as possible, due to the complexity of fathers’ parenting efficacy, the consideration of the fathers’ own attachment level and stress level was still neglected. Meanwhile, in the future, we may consider expanding the sample capacity while further optimizing the research framework by refining the study population to specific regions such as urban and rural areas, so as to explain the complex factors influencing the parenting efficacy of fathers of children with ASD in a more comprehensive and detailed way.

## Data availability statement

The raw data supporting the conclusions of this article will be made available by the authors, without undue reservation.

## Ethics statement

The studies involving humans were approved by School of Education, South China Normal University. The studies were conducted in accordance with the local legislation and institutional requirements. Written informed consent for participation was not required for this study in accordance with the national legislation and the institutional requirements.

## Author contributions

LZ: Formal analysis, Methodology, Writing – original draft, Visualization. XG: Supervision, Writing – review & editing. WH: Project administration, Validation, Writing – review & editing. LS: Investigation, Writing – original draft. XM: Investigation, Writing – original draft.
